# Catalytic oxidation of toluene, ethyl acetate and chlorobenzene over Ag/MnO_2_-cordierite molded catalyst

**DOI:** 10.1038/s41598-019-48506-5

**Published:** 2019-08-21

**Authors:** Jianan Zhu, Wenrui Zhang, Qiuping Qi, Huawei Zhang, Yaqing Zhang, Dekui Sun, Peng Liang

**Affiliations:** 1 College of Chemical and Environmental Engineering, Shandong University of Science and Technology, Qingdao, Shandong 266590 P.R. China; 2 State Key Laboratory of Coal Conversion, Institute of Coal Chemistry, Chinese Academy of Sciences, Taiyuan, Shanxi 030001 P.R. China

**Keywords:** Chemistry, Materials science

## Abstract

Multi-structured Ag/MnO_2_-cordierite molded catalysts were prepared by hydrothermal method and applied to the catalytic oxidation of VOCs. Catalytic activities of Ag/MnO_2_-cordierite were evaluated by 1000 ppm of toluene, ethyl acetate and chlorobenzene degradation respectively at the air atmosphere, and their physicochemical properties were characterized through multiple techniques containing XRD, SEM, TEM, H_2_-TPR and XPS. It is found that nanorod Ag/MnO_2_-cordierite molded catalyst showed prominent catalytic activity for VOCs decomposition and the T_90_ for toluene, ethyl acetate and chlorobenzene are 275 °C, 217 °C and 385 °C respectively under the space velocity of 10,000 h^−1^. High valence manganese oxide, more active lattice oxygen proportion and superior low-temperature reducibility were the great contributors to the high activity of the catalyst with nanorod morphology. Studies of space velocity and catalytic stability over nanorod Ag/MnO_2_-cordierite molded catalyst have confirmed the good catalytic performance, excellent mechanical strength and satisfied anti-toxicity to Cl at higher space velocity, which indicates that this molded catalyst have promise for industrial application.

## Introduction

Volatile organic compounds (VOCs) are classified as an indispensable cause for the current atmospheric pollution^1,2^. Due to the high chemical stability, toxicity and carcinogenic effect, VOCs pose serious threats to human health and ecological environment^3,4^. However, the growing emissions of VOCs owing to rapid urbanization and industrialization make it urgent to control and removal VOCs effectively^5^. Among the numerous approaches, catalytic oxidation is widely recognized as an effective and broad technique to degrade the industrial VOCs due to its low energy consumption, high purification efficiency, heat recovery and so on^6,7^. Therefore, the study of low-temperature, high-activity and anti-poisoning catalysts is a key to the catalytic oxidation in industrial process.

Many catalysts can be used for catalytic oxidation of VOCs^8–12^. Recent studies showed supported transition metal oxides catalysts such as VO_x_, MnO_x_ and WO_x_ have been applied widely and efficiently^8–10^. Specially, manganese dioxides have attracted extensive attentions because of its high selectivity and activity at low temperature^11–13^. However, some drawbacks such as poor stability, sintering and low conversion rate prevent more general applications. Articles reported presently that the incorporation of noble metals, such as Pt, Au and Ag, can promote the oxidation of VOCs and enhance the anti-toxicity of the catalyst effectively because of their higher activity for the break of C-C bond, C-H bond and C-Cl bond^14–17^. For example, the Au/Mn_2_O_3_ powdered catalyst prepared by Xie *et al*.^15^ achieved 90% conversion of toluene at the conditions of volume ratio of toluene to O_2_ of 1:400, 258 °C and space velocity of 40,000 ml/(g·h). Xia *et al*.^18^ prepared the nanotube Au-Pd/α-MnO_2_ powdered catalyst and observed the good catalytic activity for VOCs oxidation with 40–60 mesh quartz sand in a quartz tube with an inner diameter of 4 mm. The studies reported^7,12,18–20^ so far are mostly carried out by mixing the pure powdered catalyst into the millimeter-scale quartz tube reactor through incorporating inert material such as quartz sand. However, they are unsuitable for actual catalytic combustion owing to incomplete reaction between catalyst and reactants under high volume space velocity, and most of them were applied to degrade one kind of VOCs in the atmosphere with high oxygen content. There are few researches about lower temperature catalytic degradation of various VOCs and chlorinated volatile organic compound (Cl-VOCs) in actual industrial exhaust gas treatment. Moreover, catalysts which can completely destroy Cl-VOCs are restricted by high temperatures and serious deactivation in the presence of Cl^21–23^. Studies^24^ have shown that the molded catalyst can offer lower mass transfer resistance and higher diffusion efficiency, which can be a promoter for excellent catalytic activity for VOCs degradation. Cordierite^25,26^ has been widely used as carrier because of its superior mechanical stability and hydrothermal stability as well as its plasticity. For example, Mn-Co-Ce/cordierite catalyst^27^ and Cr-MnO_2_/cordierite catalyst^28^ increased the external mass transfer rate to optimize the catalytic combustion performance of Cl-VOCs. In previous work^20^, Ag/MnO_2_ powdered catalyst was prepared to degrade toluene. It has observed that excellent catalytic oxidation performance due to the multiple valence state of Mn and the better oxygen transformation between transition metal oxides in the redox reaction by Ag. Therefore, Ag/MnO_2_ may be a promising catalytic oxidation of VOCs. However, there are few studies of cordierite coated by Ag/MnO_2_ molded catalyst.

In this work, the Ag/MnO_2_ catalysts with different morphology supported on cordierite were synthesized through a liquid phase reduction method and then were used for catalytic degradation of toluene, ethyl acetate and chlorobenzene. Additionally, combined with the activity and physical property characterization results of the catalyst, the structure-activity relationship of the catalyst and the cause of the chlorine poisoning of the catalyst were investigated.

## Methods

### Catalysts preparation

The nanorod MnO_2_ and nanotube MnO_2_ were synthesized by hydrothermal method published in previous papers^12,20^, and the preparation details of the MnO_2_ precursors were described in SI-1. The Ag/MnO_2_ catalyst was synthesized by a liquid phase reduction method. Typically, quantitative MnO_2_ powder was firstly dispersed in 0.01 mol/l AgNO_3_ (in accordance with the silver loading of 2 wt%) and stirred for 2 h at room temperature to obtain a homogenous suspension. The desired volume NaBH_4_ aqueous solution (theoretical molar ratio of NaBH_4_: Ag^+^ = 2:1) was added to the suspension, followed by stirring until the reaction was finished. Next, the products were filtered, washed by deionized water and then dried at 80 °C. According to their results of morphology, the names of samples were donated as the R-Ag/MnO_2_ (nanorod Ag/MnO_2_) and T-Ag/MnO_2_ (nanotube Ag/MnO_2_). Finally, the Ag/MnO_2_ catalysts supported on cordierite (the diameter of 2 cm and height of 1 cm, 200 mesh) were manufactured using a wet impregnation method with aluminum sol as a binder. The samples were subsequently dried in an oven of 100 °C for 1 h and then calcined in a muffle furnace at 300 °C for 3 h.

### Catalytic activity tests

**Figure 1:**
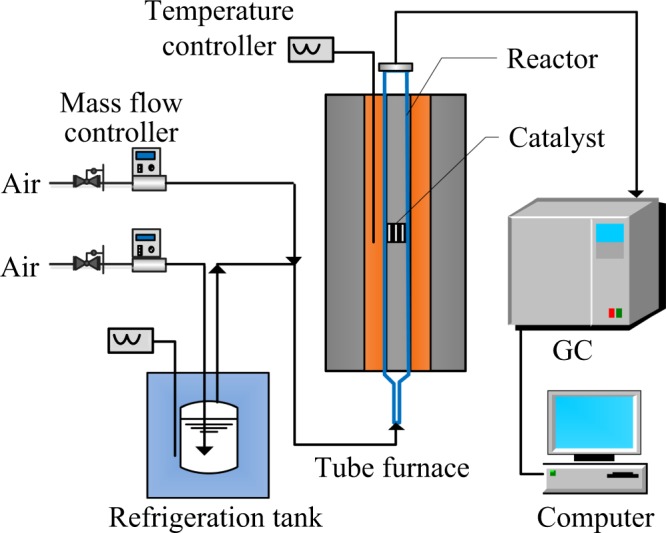
Schematic diagram of catalysts evaluation device.

Catalytic activity was implemented by a self-assembled tubular fixed bed reactor within the temperature range 80–450 °C (Fig. 1). The flow rates of each gas path were calculated based on the saturated vapor pressure of VOCs and controlled by the mass flow meters. The bubbling gas stream passed through the liquid organic matter and then merged with the equilibrium gas to obtain the desired concentration of VOCs gas, which then entered the tube furnace and reacted with the catalysts in the middle of the quartz tube. The concentration of VOCs before and after the reaction at different temperatures was obtained using the on-line gas chromatograph (GC-9790) outfitted with a flame ionization detector (FID) and a capillary column (ONLYSCI, China) of 50 m * 0.32 mm * 0.5 μm. By the way, the products of three VOCs have been identified as CO_2_ and H_2_O by clarify lime water and anhydrous copper sulfate, where HCl appeared in the oxidation products of chlorobenzene by detection of a silver nitrate solution. Meanwhile, the blank test was performed without catalysts to ensure the accuracy and reliability of experiments.

The VOCs conversion was calculated according to the following equation, where C_*In*_ and C_*Out*_ represented the concentration of VOCs in the gas before and after the reaction, respectively.

1 $$Conversion=(1-{C}_{Out}/{C}_{In})\times 100 \% $$

### Characterization of catalysts

The elemental compositions of the catalysts were measured using an Agilent 7500cx inductively coupled plasma spectrometry (ICP). The morphology of the catalysts was obtained by scanning electron microscopy (SEM, model numbers was JEOL, JSM-7800F) operating at 10 kV after precoating the samples with gold (ion sputtering apparatus, JFC1600) and transmission electron microscopy (TEM, model numbers was FEI Tecnai G2 F20) at an accelerating voltage of 200Kv. X-ray diffraction (XRD) patterns were performed by a Rigaku Utima IV X-ray diffract meter, which was equipped with Cu Kα radiation. The XRD data were collected at the range of 10 to 80° with 5°/min scan rate. The reducibility of the catalysts was measured by a Quantachrom automatic chemisorption analyzer to obtain the hydrogen temperature programmed reduction curve (H_2_-TPR). 0.05 g catalyst was placed in a mixed gas stream of 10 vol.% H_2_/Ar and then heated to 800 °C at the programmed temperature rate of 10 °C/min. The X-ray photoelectron spectroscopy (XPS) of the samples was measured using an X-ray photoelectron spectrometer equipped with an Al Kα excitation source, whose model number was Thermo ESCALAB 250Xi.

## Results and Discussion

### Catalytic oxidation of toluene, ethyl acetate and chlorobenzene

#### Catalytic activity

**Figure 2:**
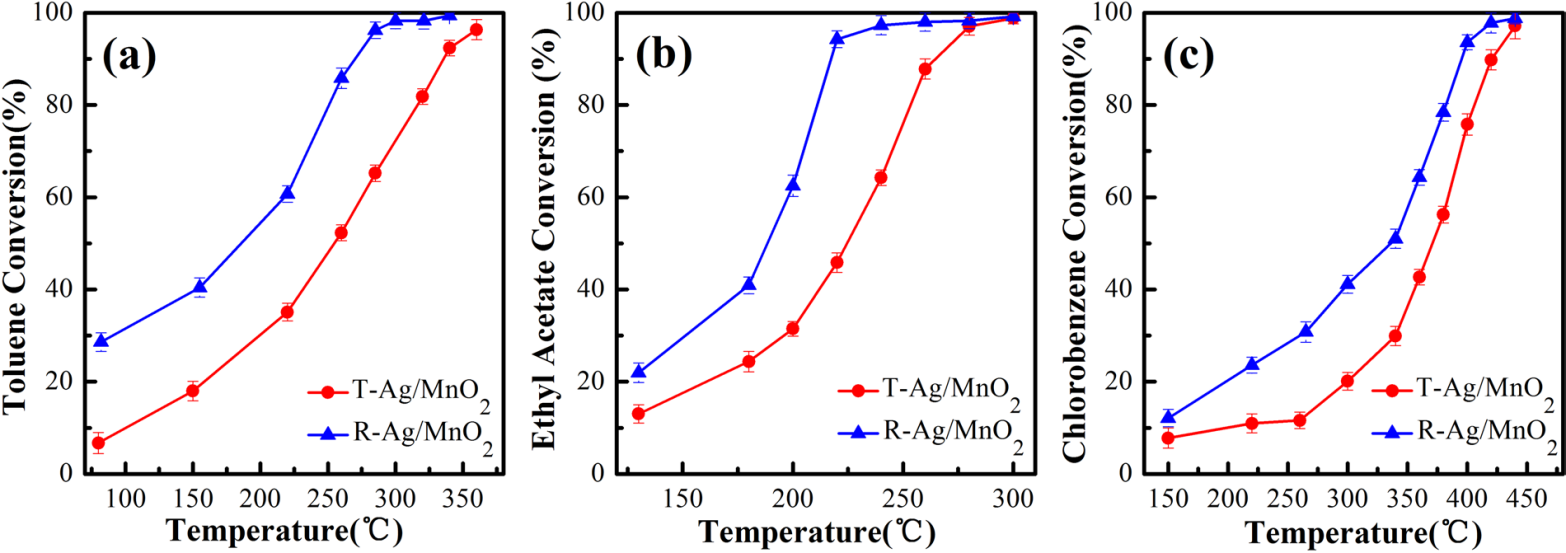
Catalytic activity of Ag/MnO_2_-cordierite catalyst for (**a**) toluene, (**b**) ethyl acetate, (**c**) chlorobenzene at different temperatures; reaction conditions: VOCs = 1000 ppm; catalyst content = 0.1 g/cm^3^; SV (space velocity) = 10,000 h^−1^.

The results of the degradation evaluation in three VOCs oxidation including toluene, ethyl acetate and chlorobenzene are shown in Fig. 2(a–c). From these, it is easy to see that the conversion of VOCs rose with the increase of the reaction temperature, and R-Ag/MnO_2_-cordierite showed better catalytic activity than T-Ag/MnO_2_-cordierite. T_90_ (Temperature corresponding to the VOCs conversion of 90%) is commonly used to evaluate and compare the catalytic activity of samples. With regard to the R-Ag/MnO_2_-cordierite, the values of T_90_ for ethyl acetate, toluene and chlorobenzene conversion were 217 °C, 275 °C and 385 °C, which were lower than those of T-Ag/MnO_2_-cordierite (260 °C, 330 °C and 420 °C), indicating that the R-Ag/MnO_2_-cordierite has better catalytic activity than T-Ag/MnO_2_-cordierite. It is worth pointing out that, with the same conversion rate, the degradation temperature required for toluene and chlorobenzene displays higher than ethyl acetate. The reason may be related to the different strength between reactants and catalyst surface. It has been found that aromatic hydrocarbon compounds composed of one saturated chain and one benzene ring have a strong interaction with the surface of catalysts, making them easier to compete with oxygen for adsorption and occupy more active sites during the reaction^29^. What’s more, comparing with other powder catalysts (MnO_x_/γ-Al_2_O_3_^21^, Pt/TiO_2_^17^ and nano-octahedra Ru/CeO_2_^30^), R-Ag/MnO_2_-cordierite molded catalyst prepared in this study requires much lower temperature for catalytic degradation of Cl-VOCs.

#### Catalysts stability

**Figure 3:**
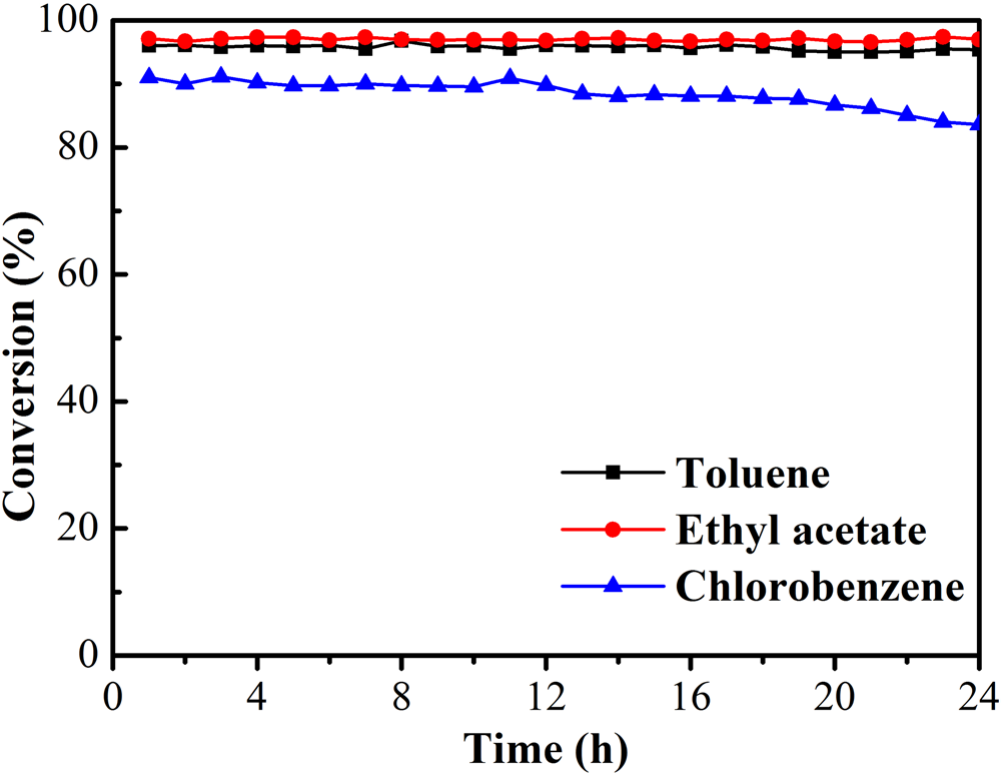
Stability tests of R-Ag/MnO_2_-cordierite for toluene, ethyl acetate and chlorobenzene; reaction conditions: temperature = 280 °C for toluene, 230 °C for ethyl acetate, 390 °C for chlorobenzene; VOCs = 1000 ppm; catalyst content = 0.1 g/cm^3^; SV = 10,000 h^−1^.

R-Ag/MnO_2_-cordierite with high catalytic activity was selected to investigate the stability of the catalysts, shown in Fig. 3. It is easy to see that the conversion of ethyl acetate and toluene remained stable for 24 h (above 95%). For the conversion of chlorobenzene, it remained 90% within first 12 h and dropped slightly for the next 12 h, which still is located at above 85% within 24 hours. This result indicates that R-Ag/MnO_2_-cordierite has great stability to VOCs and exhibits certain anti-toxicity performance to chlorobenzene. The physical properties of R-Ag/MnO_2_-cordierite reacted with chlorobenzene for 24 h (denoted as R_CB_-Ag/MnO_2_) was carried out in section 3.2.

#### Effect of space velocity

**Figure 4:**
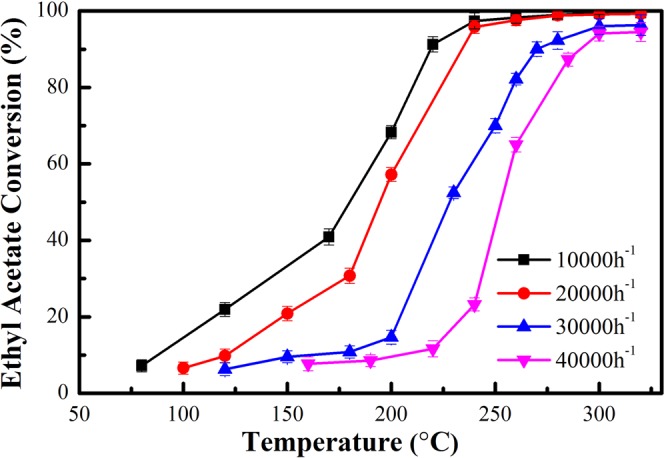
Catalytic removal efficiency of R-Ag/MnO_2_-cordierite on ethyl acetate at different space velocity; reaction conditions: VOCs = 1000 ppm; catalyst content = 0.1 g/cm^3^.

In industrial applications, space velocity has been reported to be a critical factor affecting catalytic oxidation and devices economy. Effect of space velocity on catalytic activity of R-Ag/MnO_2_-cordierite has been studied, which is shown in the Fig. 4. Obviously, the degradation effect of R-Ag/MnO_2_-cordierite is reduced slightly with the increasing of space velocity from 10,000 h^−1^ to 40,000 h^−1^, where the catalyst ignition temperature and T_90_ of ethyl acetate have risen by approximately 100 °C and 70 °C respectively at a higher SV of 40,000 h^−1^ (still lower than 300 °C, Table 1). This result indicates that the catalyst have promise for adoption by the industry due to its high activity at high space velocity.

**Table 1:** Ignition temperature and T_90_ of ethyl acetate oxidation with different space velocities.

SV/h^−1^	Ignition temperature/°C	T_90_/°C
10,000	125	217
20,000	150	230
30,000	200	265
40,000	225	287

### Physical properties of the catalysts

The elemental composition of the catalysts was measured by ICP-MS, and the results are shown in Table 2. The contents of Mn and Ag in the catalyst were about 42.6 wt% and 1.9 wt%, respectively. The Ag content coincides with the theoretical ratio of 2 wt% after deducting the aluminum sol component, which means that the noble metal Ag has been loaded into MnO_2_ successfully.

**Table 2:** Catalysts element components.

Samples	Mn/wt%	Ag/wt%	Al/wt%	Ag/wt% (Al deducted)
T-Ag/MnO_2_	42.24	1.54	8.13	1.93
R-Ag/MnO_2_	41.97	1.53	7.97	1.91
R_CB_-Ag/MnO_2_	42.66	1.51	8.51	1.89

**Figure 5:**
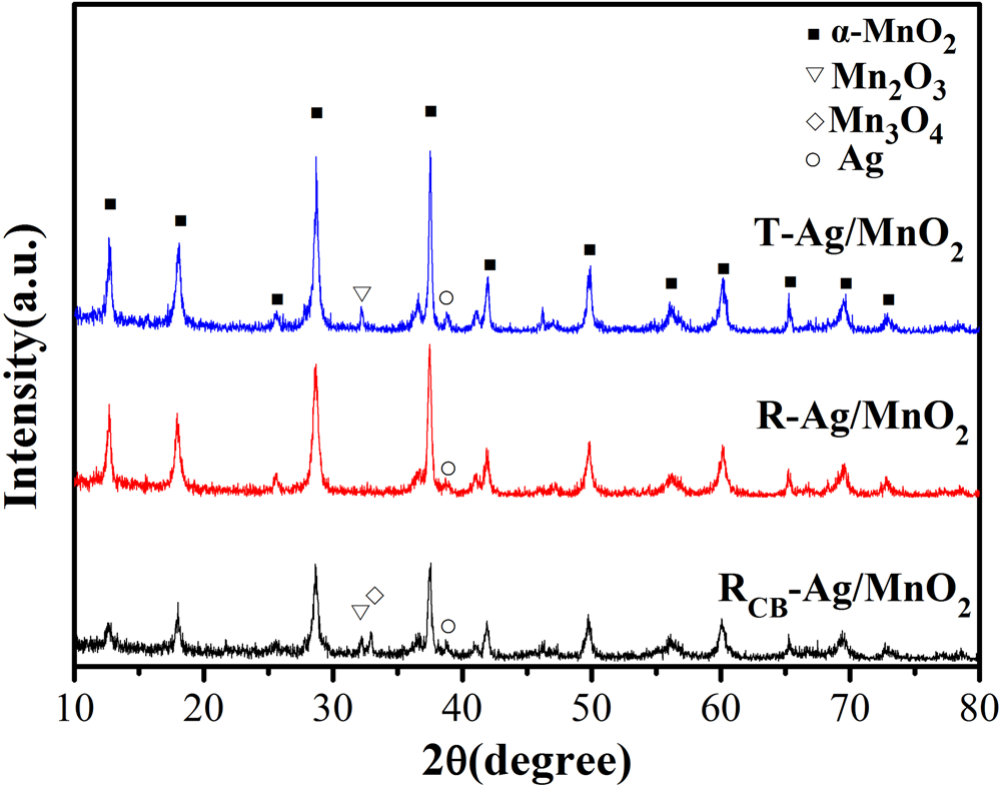
XRD patterns of Ag/MnO_2_ catalysts.

Figure 5 is the XRD patterns of the three catalysts. The presence of aluminum sol on the oxide supports had no effect on crystal formation of the catalysts (shown in SI-2). It is found that all samples in the 2θ range of 10–80° exhibit characteristic diffraction peaks of α-MnO_2_ (ICSD PDF 44–0141) and Ag (111) crystal plane at 38.1° (PDF#65-2871)^15^. An additional diffraction peak of T-Ag/MnO_2_ is observed at 32.5° assigning to Mn_2_O_3_ comparing with R-Ag/MnO_2_, which was probably due to the addition of HCl during preparation. Furthermore, the diffraction peaks of Mn_2_O_3_ and Mn_3_O_4_ (32.5° and 33.0°^14^) appeared and the peak intensity of α-MnO_2_ significantly weakened in the pattern of R_CB_-Ag/MnO_2_. This result was related with the structural transformation of α-MnO_2_ to Mn_2_O_3_ and Mn_3_O_4_, which may take place in the process of catalytic degradation of chlorobenzene. Related studies^12^ have shown that manganese oxides exhibit different activity in some benzenes catalytic oxidation reaction. Higher oxidation state of Mn species, namely MnO_2_, is preferable for oxidation reactions over Mn_2_O_3_ and Mn_3_O_4_. Combined with results of 3.1.1, it is reasonable to suggest that the catalytic activity of R-Ag/MnO_2_-cordierite with pure α-MnO_2_ is superior to that of T-Ag/MnO_2_-cordierite with mixed α-MnO_2_ and Mn_2_O_3_. Moreover, it was assumed that the formation of lower manganese oxides was responsible of a decrease in activity of R-Ag/MnO_2_-cordierite after chlorobenzene catalytic oxidation.

**Figure 6:**
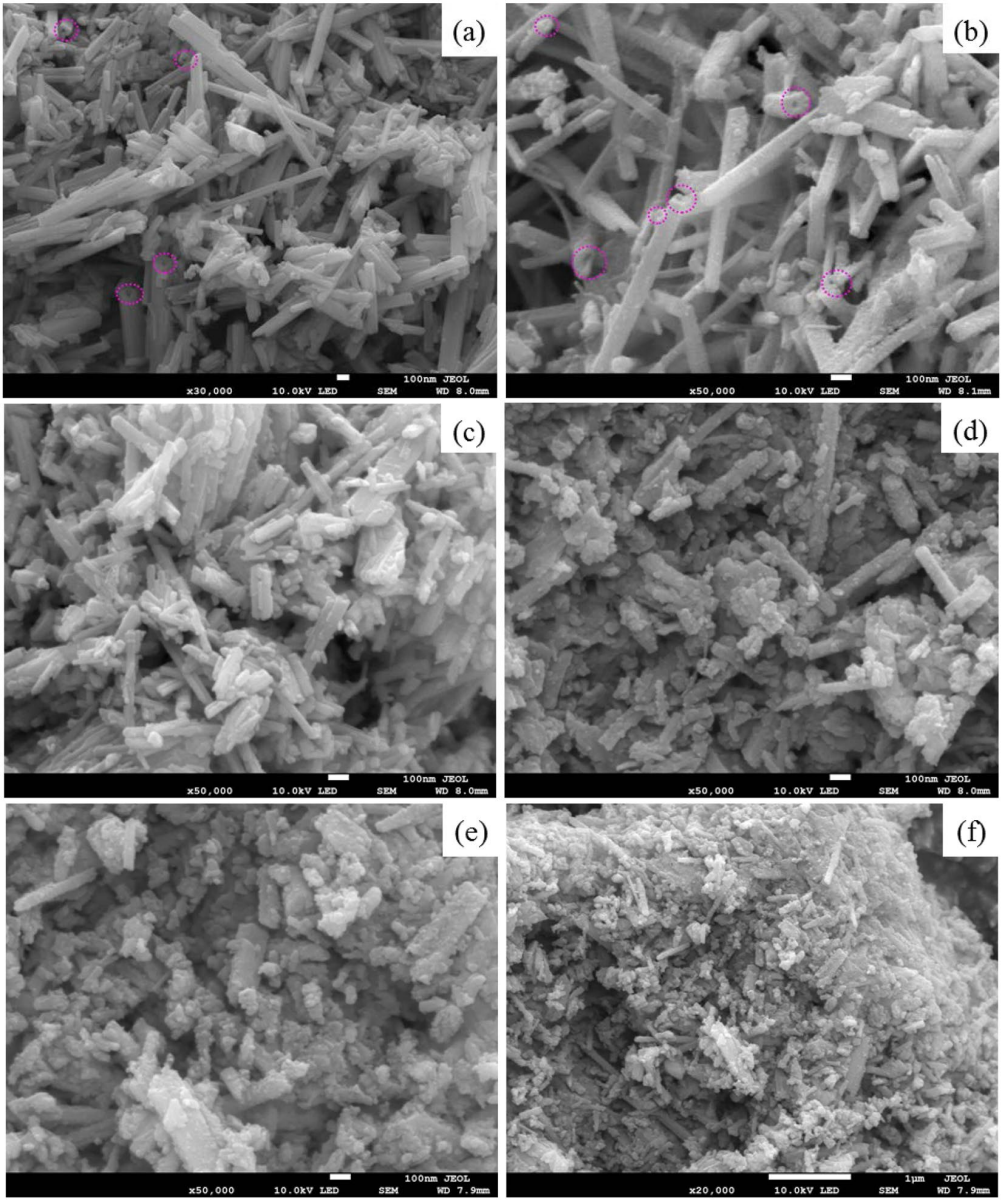
SEM image of (**a**) T-Ag/MnO_2_, (**b**) T-Ag/MnO_2_ with aluminum sol, (**c**) R-Ag/MnO_2_, (**d**) R-Ag/MnO_2_ with aluminum sol, (**e**,**f**) R_CB_-Ag/MnO_2_ with aluminum sol.

The SEM images of each sample are shown in the Fig. 6. Nanotube and nanorod morphology were observed in Fig. 6(b,d), and no clear differences in size or shape was detected in Fig. 6(a,b) or Fig. 6(c,d), indicating that the existence of aluminum sol has no influence in Ag/MnO_2_ catalyst morphology. It was also found that there was little change between R-Ag/MnO_2_ (Fig. 6(d)) and R_CB_-Ag/MnO_2_ (Fig. 6(f)), except that the nanorod length of R_CB_-Ag/MnO_2_was shorter.

**Figure 7:**
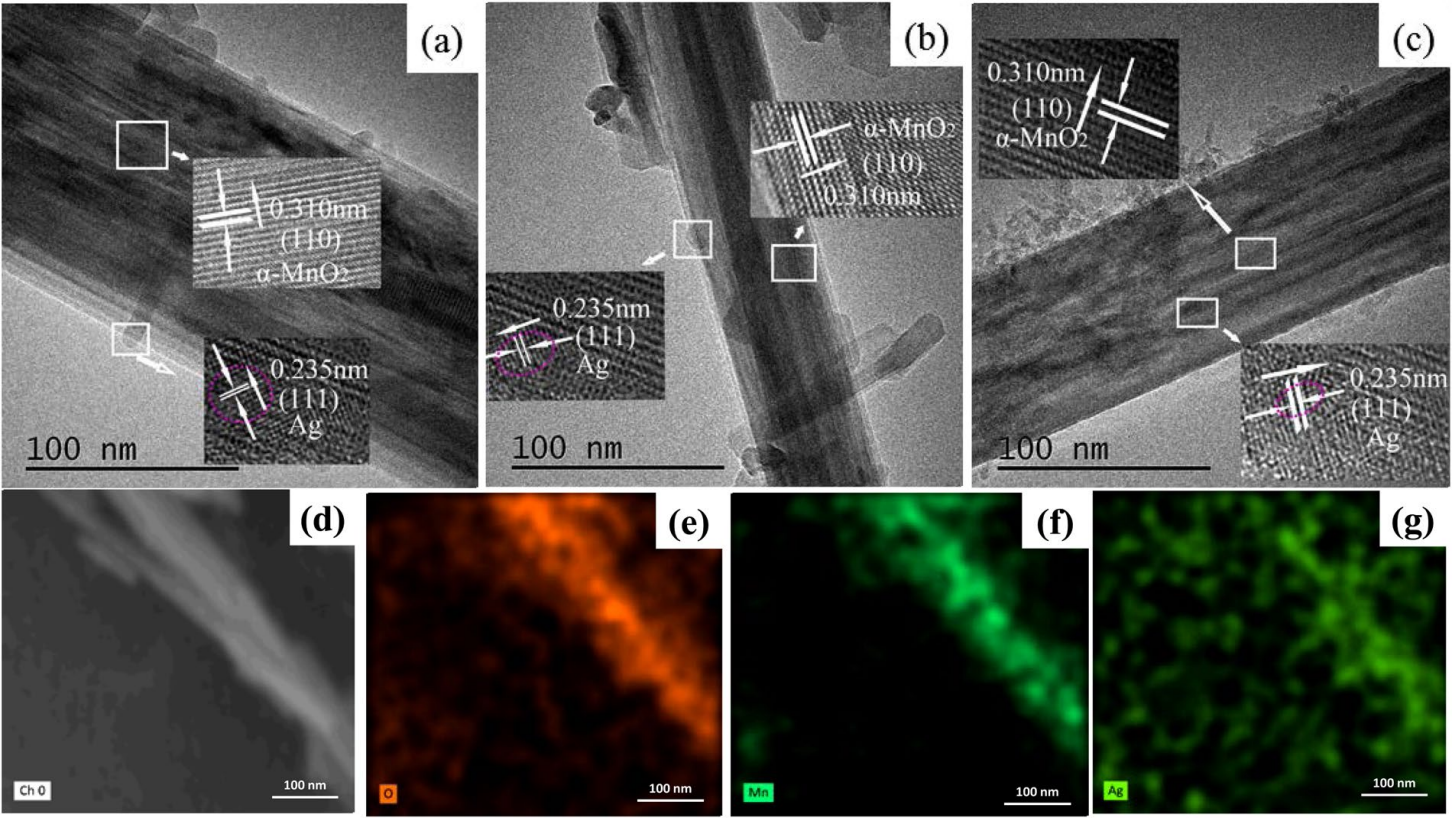
TEM micrograph of the (**a**) T-Ag/MnO_2_, (**b**) R-Ag/MnO_2_, (**c**) R_CB_-Ag/MnO_2_ with aluminum sol; (**d**–**g**) SEM element mapping images of the R-Ag/MnO_2_.

Figure 7 reveals TEM images of Ag/MnO_2_ samples and SEM element mapping images of the R-Ag/MnO_2_. The Ag loaded on the catalyst with good dispersion (about 5 nm) and the Ag/MnO_2_ catalysts retained fine crystalline structure. The lattice spacing of the (110) and (111) crystal plane was 0.310 nm and 0.235 nm, respectively, which is in line with the corresponding values (0.310 nm) of standard α-MnO_2_ sample (JCPDS PDF 72–1982) and (0.236 nm) of standard Ag sample (JCPDS PDF 04–0783). It has been observed that rod-like Ag/MnO_2_ catalyst exhibits the higher VOCs conversion, whereas T-Ag/MnO_2_ shows relatively low catalytic activity. This difference in catalytic activity is attributed to the catalyst morphology. To our knowledge, catalysts with different morphology can expose different active or specific energy crystal faces selectively and some of them with special morphology might promote the better dispersion of active components. That can be contributed greatly to improve catalytic reactivity, selectivity and stability^12^. For this study, the R-Ag/MnO_2_ could be more favorable to increase the effective activated spot and improve the catalytic activity better than T-Ag/MnO_2_. As reported, the degradation of chlorobenzene calls for higher requirements for the stability of catalysts^23^. The morphology of R-Ag/MnO_2_ and the crystallinity of α-MnO_2_ are no variation after degrading Cl-VOCs at high space velocity, indicating that R-Ag/MnO_2_ catalyst has excellent mechanical strength and stability in this study.

**Figure 8:**
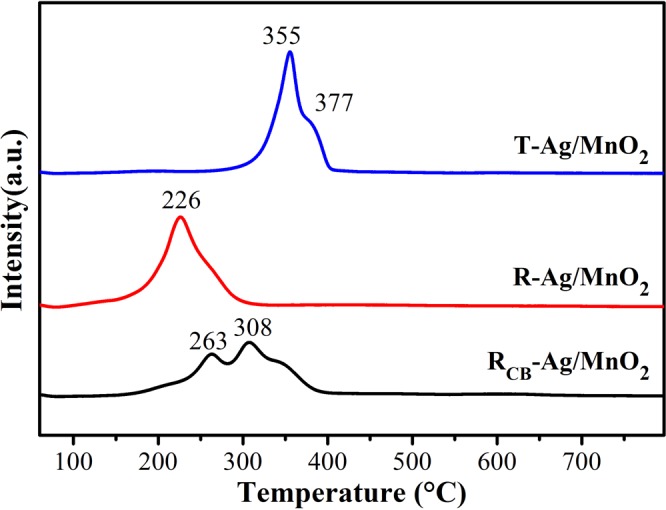
H_2_-TPR profiles of Ag/MnO_2_ catalysts.

H_2_-TPR curves of Ag/MnO_2_ catalysts are shown in Fig. 8. A large broad and strong peak at about 226 °C of the R-Ag/MnO_2_ pattern could be considered as the transformation from high-valent manganese ions to lower valence state. For T-Ag/MnO_2_ catalyst, the reduction peak and a shoulder peak shifted to the higher temperatures at 355 °C and 377 °C compared to R-Ag/MnO_2_. This indicates that R-Ag/MnO_2_ possesses better low temperature reduction performance than T-Ag/MnO_2_. After catalytic oxidation of chlorobenzene, the main reduction peaks of Mn species for R_CB_-Ag/MnO_2_ are shifted to higher temperature and could be regarded as two peaks at about 263 °C and 308 °C, which means the reduced reducibility may be one reason of the slight decrease in catalytic activity (combined with results of 3.1.2). In the light of previous results^12,31^, the transformation process of MnO_2_ can be reasonably interpreted as two steps: (1) MnO_2_→Mn_2_O_3_ corresponding to peaks at 226 °C, 263 °C and 355 °C, (2) Mn_2_O_3_→Mn_3_O_4_ corresponding to peaks at 308 °C and 377 °C, respectively. As mentioned previously^13^, the presence of more high-valence manganese is beneficial to the low-temperature catalytic oxidation of VOCs. Combined with the results of XRD, it can be inferred that R-Ag/MnO_2_-cordierite with high valence state of manganese may be the reasons for the strong reducibility and higher catalytic activity^32^.

**Figure 9:**
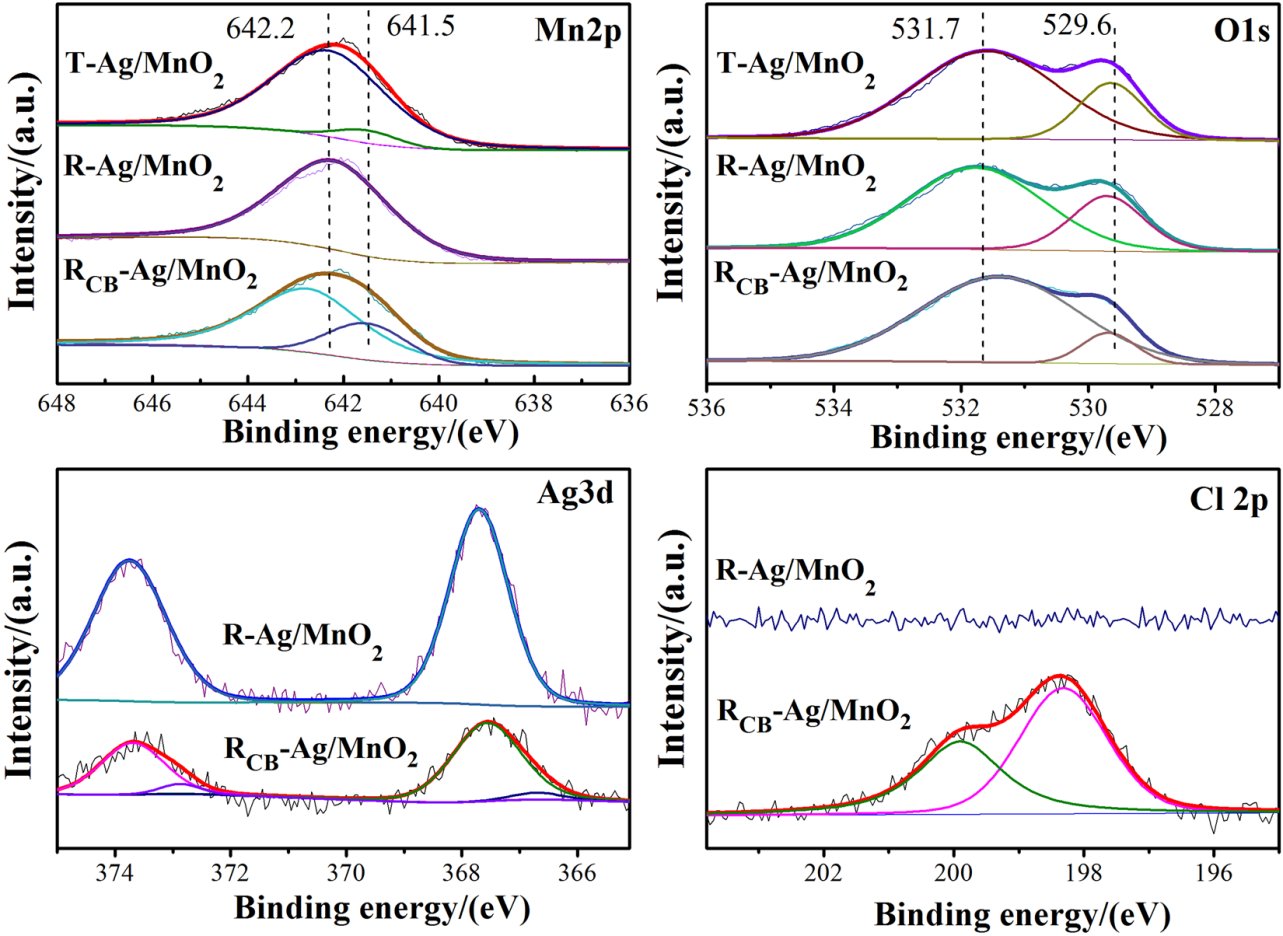
Mn 2p, O 1s, Ag 3d and Cl 2p XPS spectrum of Ag/MnO_2_.

Figure 9 illustrates the Mn 2p, O 1s, Ag 3d and Cl 2p XPS spectra of Ag/MnO_2_ catalysts. The Mn 2p peaks appeared at 642.2 eV and 641.5 eV, which are attributed to the Mn^4+^ and Mn^3+^, respectively^13,33,34^. Compared with R-Ag/MnO_2_, the chemical valence of Mn of T-Ag/MnO_2_ is mainly in Mn^4+^ oxidation state and a small quantity of Mn^3+^ co-existed. It has been verified that the higher extent of the Mn^4+^ favors the redox reactions and gives a boost to the catalytic performance of VOCs degradation^35^, as well as the presence of element Mn plays a facilitating role in the production of oxygen vacancies and the enhancement of oxygen mobility^36^. From Table 3, it could be found that the molar ratio of surface Mn^4+^ of R-Ag/MnO_2_ is higher comparing with T-Ag/MnO_2_, which gives rise to the fact that the nanorod Ag/MnO_2_ molded catalyst exhibited good catalytic activity for VOCs. In addition, Mn^4+^/Mn value (0.75) of R_CB_-Ag/MnO_2_ was markedly reduced because of the reduction of MnO_2_ to Mn_2_O_3_ during catalytic oxidation of chlorobenzene. The results were also consistent with the aforementioned XRD and H_2_-TPR. According to the XPS spectrum of O1s, the peak at 529.6 could be ascribed to the lattice oxygen (O_latt_), while the peak at 531.7 eV could be ascribed to the adsorbed oxygen (O_ads_)^14^. As reported^20,37^, the atomic ratio of the O_latt_/O_ads_ could influence the catalytic oxidation of VOCs. Different oxygen species content in each catalyst was obtained according to XPS characterization (shown in the Table 3). The amount of O_latt_/O_ads_ of R-Ag/MnO_2_ is much higher than that of T-Ag/MnO_2_, suggesting that rod-like Ag/MnO_2_ may be beneficial to the lattice oxygen migration, which should administer to enhance the performance of R-Ag/MnO_2_ for the catalytic oxidation of VOCs. Besides, the O_latt_/O_ads_ value of R_CB_-Ag/MnO_2_ is significantly much less in comparison with that of R-Ag/MnO_2_. The result can be attributed to the fact that lattice oxygen facilitates the VOCs oxidation reaction at low temperatures.

**Table 3:** Contents of different oxygen species and manganese.

Sample	BE (eV)		O_latt_/O_ads_	Mn^4+^/Mn
	Lattice oxygen	Absorbed oxygen		
T-Ag/MnO_2_	529.6	531.6	0.47	0.93
R-Ag/MnO_2_	529.7	531.7	0.61	1.00
R_CB_-Ag/MnO_2_	529.7	531.4	0.14	0.75

The peaks at 373.8 eV and 367.8 eV according to the XPS spectrum of Ag 3d are assigned to the 3d_5/2_ and 3d_3/2_ of Ag, respectively, while the other two peaks at 372.9 eV and 366.8 eV are attributed to silver 3d_3/2_ and 3d_5/2_ of AgCl^38–42^. Apparently, the intensity of Ag 3d peak of R-Ag/MnO_2_ was significantly reduced after catalytic oxidation with chlorobenzene. Furthermore, the Cl 2p XPS spectrum of R_CB_-Ag/MnO_2_ catalyst reveals two peaks at 199.9 eV and 198.3 eV, which represent Cl 2p_3/2_ and Cl 2p_1/2_ binding energy separately^39–41^. According to the results of stability test, it is speculated that small amounts of AgCl are formed and accumulated over the catalyst surface to inactive catalyst, which may be the reason for the slight decrease in activity of R-Ag/MnO_2_-cordierite during the long-term reaction with chlorobenzene.

From all the characterization results, the R-Ag/MnO_2_-cordierite molded catalyst shows higher activity to remove VOCs than T-Ag/MnO_2_-cordierite molded catalyst due to several reasons. According to the XRD, H_2_-TPR and XPS, it’s easy to find that manganese with a high valence state could carry more lattice oxygen and possess stronger reducibility contributed to higher catalytic activity of the nanorod Ag/MnO_2_. Besides, it also can be seen from the results of SEM and TEM that the rod-like morphology and fine crystalline structure of catalysts determine the excellent mechanical strength and stability, thus leading to the high catalytic stability at higher space velocity. Last but not least, the presence of silver could accelerate the lattice oxygen migration on MnO_2_ during VOCs oxidation process, which should administer to enhance the performance of R-Ag/MnO_2_ for the catalytic oxidation of VOCs.

## Conclusions

In this study, Ag/MnO_2_ with nanorod and nanotube morphology supported on honeycomb cordierite catalysts were successfully synthesized using a liquid phase reduction method. Various characterization techniques were used to investigate their physicochemical properties and the catalytic oxidation mechanism of toluene, ethyl acetate and chlorobenzene. The Ag/MnO_2_-cordierite molded catalyst with nanorod morphology performs much better than other catalysts because of high valence manganese oxide, more active lattice oxygen content and preferred low-temperature reducibility. Experimental results show that the nanorod Ag/MnO_2_-cordierite exhibits T_90_ for toluene, ethyl acetate and chlorobenzene conversion at 275 °C, 217 °C and 385 °C respectively under the space velocity of 10,000 h^−1^. For the different space velocity and catalytic stability test, nanorod Ag/MnO_2_-cordierite show best mechanical strength, better catalytic durability and satisfied anti-toxicity to chlorobenzene at higher space velocity. These results reveal that the molded catalyst is characterized by lower cost, higher activity and stability as well as anti-chlorine poisoning ability. It has industrial application prospects in the oxidation of different kinds of VOCs.

## Supplementary information

Supporting information
