# Supplementary material for: Catalytic oxidation of toluene, ethyl acetate and chlorobenzene over Ag/MnO2-cordierite molded catalyst
Source: Sci Rep. 2019 Aug 21;9:12162. doi: 10.1038/s41598-019-48506-5 (PMC6704122; doi:10.1038/s41598-019-48506-5)
Supplement: Supplementary file 1 — Supporting information [file 41598_2019_48506_MOESM1_ESM.pdf]

Catalytic oxidation of toluene, ethyl acetate and chlorobenzene over Ag/MnO_2-cordierite molded catalyst

Jianan Zhu[1], Wenrui Zhang[1,*], Qiuping Qi[1], Huawei Zhang[1], Yaqing Zhang[1], Dekui Sun[2], Peng Liang[1,*]

[1] College of Chemical and Environmental Engineering, Shandong University of Science and Technology, Qingdao, Shandong 266590, P.R. China

[2] State Key Laboratory of Coal Conversion, Institute of Coal Chemistry, Chinese Academy of Sciences, Taiyuan, Shanxi 030001, P.R. China

[*] Corresponding authors:

Wenrui Zhang:*Tel.:+86 15253263237; Fax: +86 532 86057718;

E-mail:wenrui.mao@163.com

Peng Liang:*Tel.:+86 13678890728; Fax: +86 532 86057718;

E-mail:liangpeng202@hotmail.com

SI-1:

The preparation details of the nanorod MnO_2 and nanotube MnO_2 precursors.

SI-2:

XRD patterns of Ag/MnO_2 with and without aluminum sol.

SI-1

The preparation details of the nanorod MnO_2 and nanotube MnO_2 precursors:

The nanorod MnO_2 was prepared with the following properties: Amounts of $KMnO_4$ and $MnSO_4$•H_2O (molar ratio of 8:3) were dissolved in 80 ml of distilled water and kept stirring at room temperature for 1 h. The mixed solution was then poured into the 100 ml stainless steel autoclave and reacted at 160 ℃ for 12 h within an oven.

The nanotube MnO_2 was prepared with the following properties: 1.32 ml of HCl (37%) was added dropwise to the $KMnO_4$ aqueous solution (0.63 g of $KMnO_4$ in 80 ml deionized water) with vigorous stirring at 25 ℃. After stirring for 1 h, the mixture solution was poured into the 100 ml stainless steel autoclave and reacted at 160 ℃ for 12 h within an oven.

The above two precursors were separately filtered, washed with distilled water and placed in an oven at 80 ℃ for 3 h. The solid was followed to be calcined at the rate of 5 ℃/min to 500 ℃ and equilibrated at this temperature for 3 h, then obtaining the rod- like and tube-like MnO_2 samples.

SI-2

XRD patterns of Ag/MnO_2 with and without aluminum sol

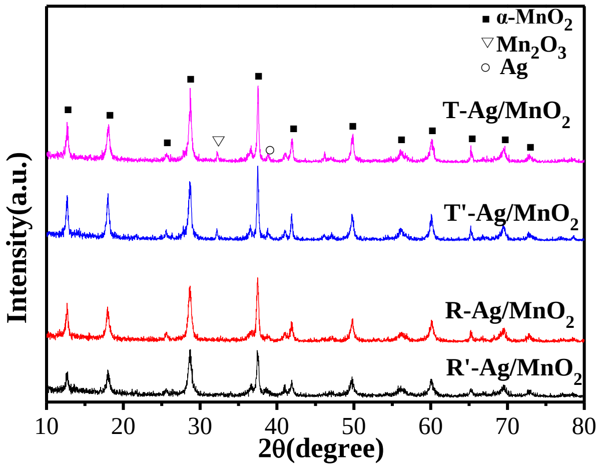

Fig. SI.2. XRD patterns of Ag/MnO_2 with and without aluminum sol with aluminum sol: R-Ag/MnO_2 and T-Ag/MnO_2; without aluminum sol: R'-Ag/MnO_2 and T'-Ag/MnO_2

XRD patterns of R-Ag/MnO_2 and T-Ag/MnO_2 with and without the aluminum sol are shown in Fig. SI.2. There is no obvious difference in position and intensity of the diffraction peaks of α-MnO_2, Mn_2O_3 and Ag between R-Ag/MnO_2 and R'-Ag/MnO_2, T-Ag/MnO_2 and T'-Ag/MnO_2.
